# Diffuse alveolar haemorrhage associated with subsequent development of ANCA positivity and emphysema in three young adults

**DOI:** 10.1186/s12890-019-0947-y

**Published:** 2019-10-24

**Authors:** Anna Stainer, Alex Rice, Anand Devaraj, Joseph Luke Barnett, Jacqueline Donovan, Maria Kokosi, Andrew Gordon Nicholson, Tom Cairns, Athol Umfrey Wells, Elisabetta Augusta Renzoni

**Affiliations:** 1 Interstitial Lung Disease Unit, Royal Brompton Hospital, London, UK; 2 Department of Histopathology, Royal Brompton Hospital, London, UK; 3 Department of Radiology, Royal Brompton Hospital, London, UK; 4 Department of Clinical Biochemistry, Royal Brompton Hospital, London, UK; 5 National Heart and Lung Institute, Imperial College London, London, UK; 6 Imperial College Healthcare NHS Trust, London, UK

**Keywords:** ANCA, Pulmonary haemosiderosis, Haemoptysis, Pulmonary haemorrage, Pulmonary vasculitis, Emphysema, AAV ANCA associated vasculitis.

## Abstract

**Background:**

Diffuse alveolar haemorrhage (DAH) is characterized by the diffuse accumulation of red blood cells within the alveoli, presence of ground glass opacities and/or consolidation on computed tomography (CT). Aside from identifiable non-immune causes, DAH is classically subdivided into idiopathic (idiopathic pulmonary haemosiderosis, IPH) and autoimmune DAH. Here we describe three cases presenting with recurrent pulmonary haemorrhage, initially classified as IPH, who, several years after first presentation, develop anti myeloperoxidase antibodies (MPO) positivity, emphysema on CT and, in one case, renal involvement.

**Case presentation:**

Patient 1 was diagnosed with IPH aged 14. Her disease remained poorly controlled despite immunosuppression, although ANCA remained negative over the years. Nineteen years from initial presentation, she developed MPO-ANCA positive antibodies and mild renal impairment. She was treated with Rituximab with good response. From first presentation, the chest CT was consistently characterized by diffuse ground-glass opacities and interlobular septal thickening. Ten years later, cystic opacities consistent with emphysema, with a striking peribronchovascular distribution, developed. Patient 2 was diagnosed with IPH aged 32. He was treated with corticosteroids and methotrexate, with fluctuating response. At 11 years from initial presentation, MPO-ANCA positivity was identified, and emphysema with a peribronchovascular distribution was observed on CT, with subsequent significant increase in extent. Patient 3 was diagnosed with IPH at the age of seven, and had recurrent episodes of haemoptysis of varying degree of severity, treated with intermittent courses of corticosteroids until age 11, when he was intubated due to severe DAH. Eight years after the diagnosis emphysematous changes were noted on CT and MPO-ANCA positivity developed for the first time 11 years after initial diagnosis.

**Conclusions:**

We believe these three cases highlight: 1) the possibility of development of ANCA positivity several years down the line from first DAH presentation 2) the possibility that DAH may lead to cystic/emphysematous changes with peribronchovascular distribution on CT. Moreover, the need for ongoing immunosuppressive treatment and the development of emphysema, emphasize a possible role played by autoimmune phenomena, even when DAH is initially diagnosed as “idiopathic”. Further studies are required to better understand the relationship between DAH, ANCA positivity and development of emphysema.

## Background

Diffuse alveolar haemorrhage (DAH) is characterized by intra-alveolar accumulation of red blood cells, with diffuse ground glass opacities and/or consolidation on chest high-resolution computed tomography (HRCT). The clinical spectrum of DAH ranges from incidental findings on imaging and/or bronchoalveolar lavage (BAL) in asymptomatic patients, to life-threatening acute respiratory failure. Histologically, DAH is characterized by the presence of hemosiderin-laden macrophages, fibrin deposition, type II pneumocyte hyperplasia, organizing pneumonia and acute inflammation. When present, capillaritis is associated with neutrophilic interstitial infiltration and disruption of the alveolar wall, although these changes can be subtle and difficult to detect [1].

DAH etiology is wide, including immunological and non-immunological causes. Among immunological causes of DAH, systemic vasculitides are one of the most frequent, particularly ANCA associated vasculitis (AAV). If no known cause or association can be found, DAH is classified as idiopathic pulmonary hemosiderosis (IPH) [2].

We describe three cases presenting with recurrent pulmonary haemorrhage, who develop anti myeloperoxidase antibodies (MPO) positivity and radiologically, cystic areas resembling emphysema many years after their first presentation.

## Case presentation

### Patient 1

**Fig. 1:**
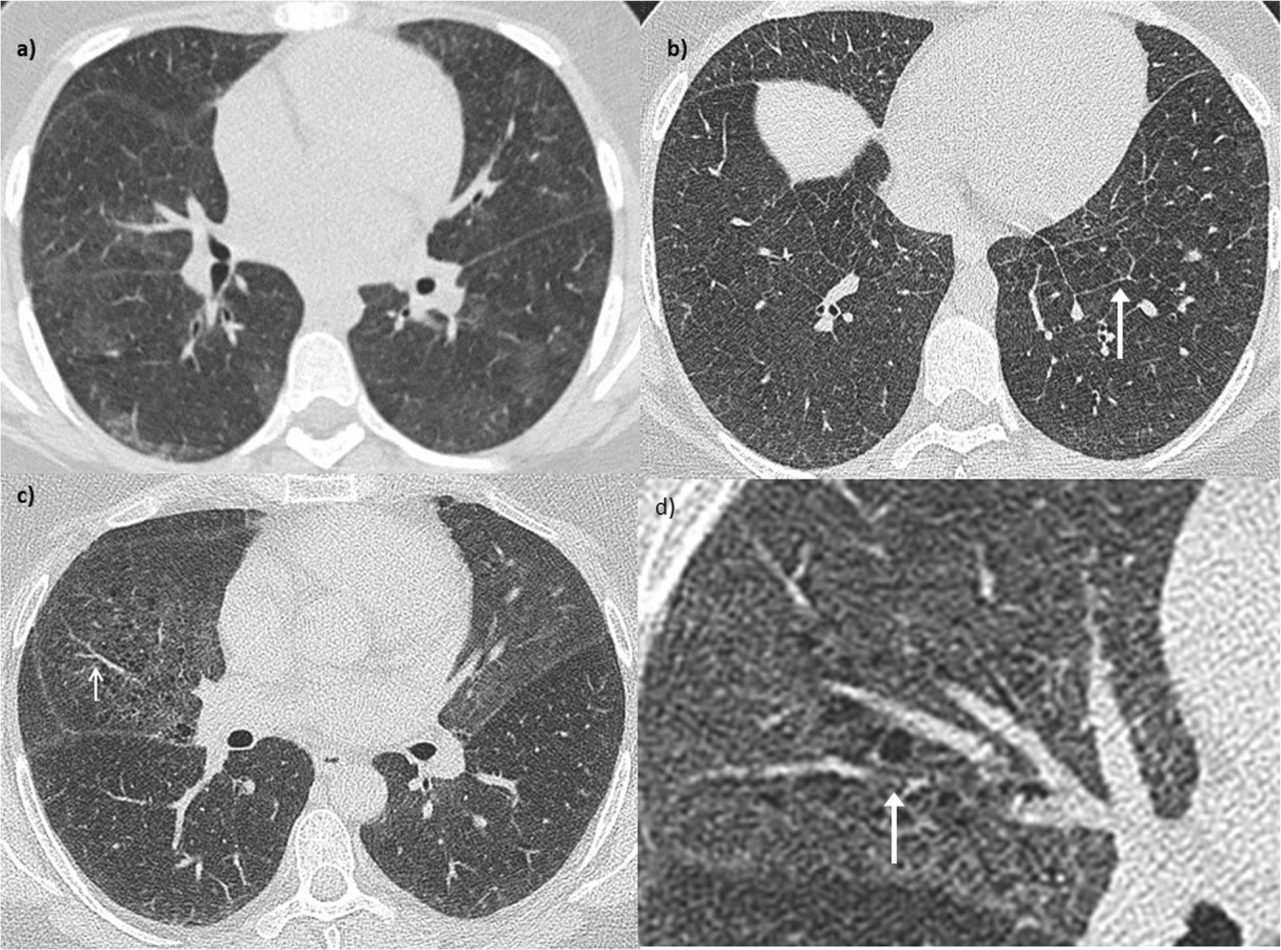
Patient 1. Radiologic evolution. Axial CT images of the lungs **a**) At 14 years old (the time of presentation), demonstrating a widespread ground glass infiltrate which has a geographic configuration; consisting of sharp demarcation between the infiltrate and normal lung. No interlobular septal thickening is evident. **b**) At 21 years old, the ground glass infiltrate appears more diffuse, and smooth interlobular septal thickening is present (arrow). **c**) At 31 years old, new emphysema is evident, in addition to a persisting ground glass infiltrate. **d**) Magnification view of C; the emphysema is seen to track along pulmonary vessels (arrow), indicating interstitial emphysema

**Fig. 2:**
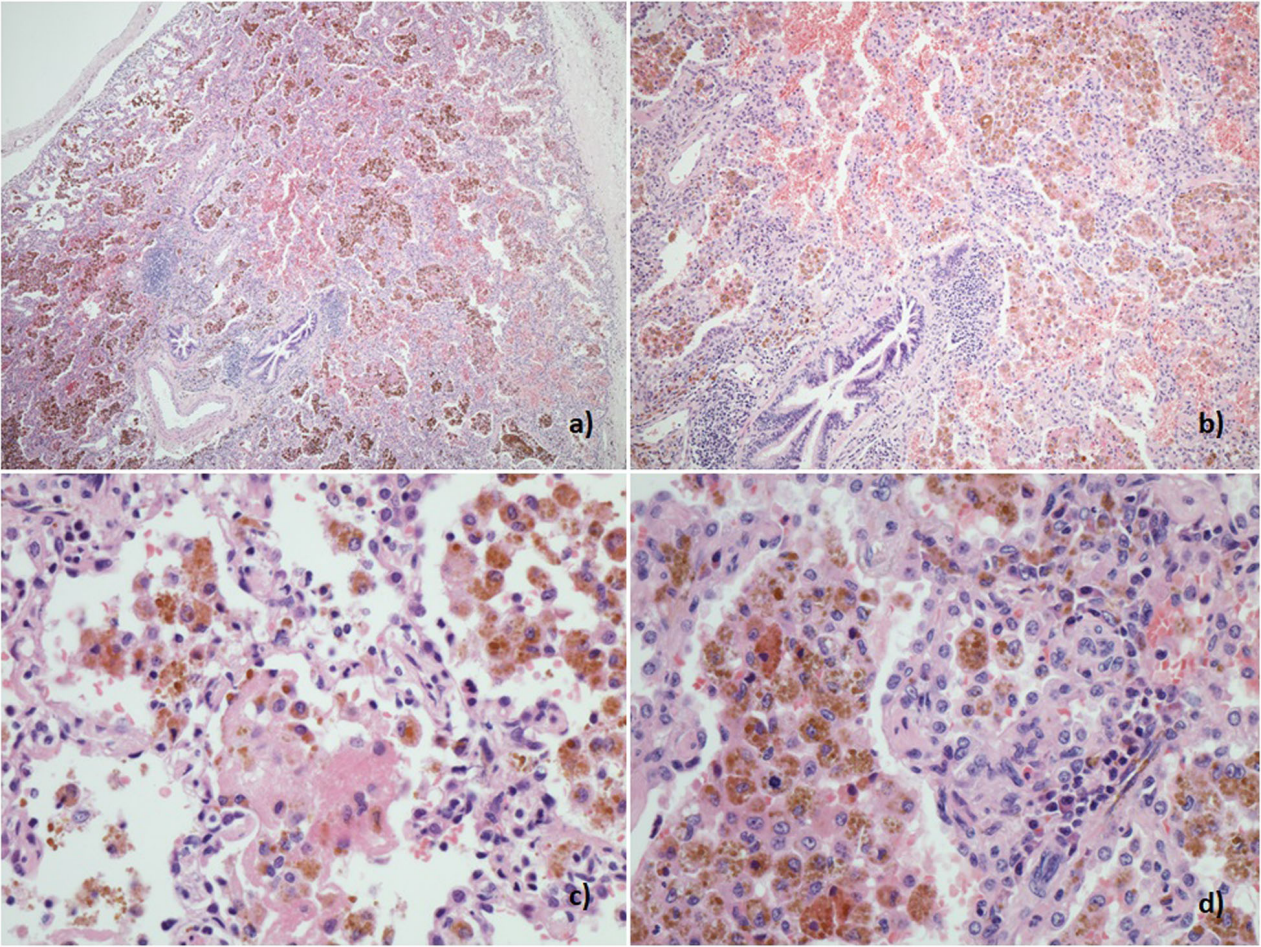
Patient 1. Histological sample, lung biopsy. **a** Marked accumulation of haemosiderin laden macrophages in alveolar spaces (H&E × 40). **b** Mild chronic bronchiolitis with small lymphoid aggregates (H&E × 100) **c** Focal alveolar fibrin accumulation and mild chronic interstitial inflammation (H&E × 400) **d** Mild chronic interstitial inflammation and reactive type 2 pneumocyte hyperplasia (H&E × 400)

A 14 year-old young woman with lethargy, exertional dyspnea, microcytic hypochromic anemia, persistent cough, and multiple episodes of haemoptysis was referred to the Royal Brompton Hospital (RBH) respiratory paediatric service. Lung function was characterized by a mild restrictive pattern (forced vital capacity (FVC) 73%, forced expiratory volume in 1 s (FEV1) 77.5%, total lung capacity (TLC) 77%, carbon monoxide transfer factor (TLCO) 85% and transfer coefficient (KCO) 110%). HRCT revealed widespread ground glass opacification throughout both lungs (Fig. 1-a). A bronchoalveolar lavage (BAL) revealed increasingly haemorrhagic returns and abundant haemosiderin laden macrophages. A surgical lung biopsy showed findings consistent with DAH (Fig. 2). In the absence of identifiable associations, the patient was diagnosed with IPH and started on hydroxychloroquine. However, her disease remained inadequately controlled with frequent flares over the years, with poor compliance a possible contributor.

Aged 21, she was admitted to the RBH adult interstitial lung disease (ILD) unit with worsening breathlessness, frequent “infectious” exacerbations, and daily small volume haemoptysis. At the time, she denied any connective tissue disease (CTD) symptoms. She only had an intermittent smoking history over the previous 3 years, totaling one pack-year. Autoimmune screen was negative. Although atypical non-specific anti-neutrophil cytoplasmic antibody (ANCA) staining was found by indirect immunofluorescence [NOVA-lite, INOVA Diagnostics Inc., San Diego, USA]), both MPO and anti-proteinase 3 (PR3) were negative (fluorescence enzyme immunoassay on Immunocap 250 analyser [Thermo Scientific ImmunoDiagnostics, Milton Keynes, UK]). A chest HRCT again revealed diffuse ground glass opacification, in addition to interlobular septal thickening (Fig. 1 b). On lung function, she had developed mild obstruction and TLCO had significantly worsened (48%), without echocardiographic evidence of pulmonary hypertension. Over subsequent years, varying doses of oral and intravenous corticosteroids were used. Both Azathioprine and Methotrexate were not tolerated. Mycophenolate mofetil achieved control of the haemoptysis for a number of years, although with the need of ongoing prednisolone associated with significant weight gain. In her late twenties, the intercurrent haemoptysis recurred. Mild renal impairment was identified with microscopic haematuria but no red blood cell casts.

At the age of 32, the patient developed p-ANCA, with MPO positivity of 3.5 u/ml, confirmed on a second sample (27 u/ml). On HRCT, in addition to the known ground-glass opacities, new emphysema was evident (Fig. 1 c-d), distributed alongside pulmonary vessels, in keeping with interstitital emphysema. She was started on intravenous cyclophosphamide with partial response followed by Rituximab which was continued at six monthly intervals. At her last follow up visit, 10 months after initiation of Rituximab, she reported improved breathlessness and no further episodes of haemoptysis. Renal function was stable although slightly abnormal and lung function remained stable with mild obstruction and disproportionate reduction in DLCO and KCO, suggestive of the development of emphysema.

### Patient 2

**Fig. 3:**
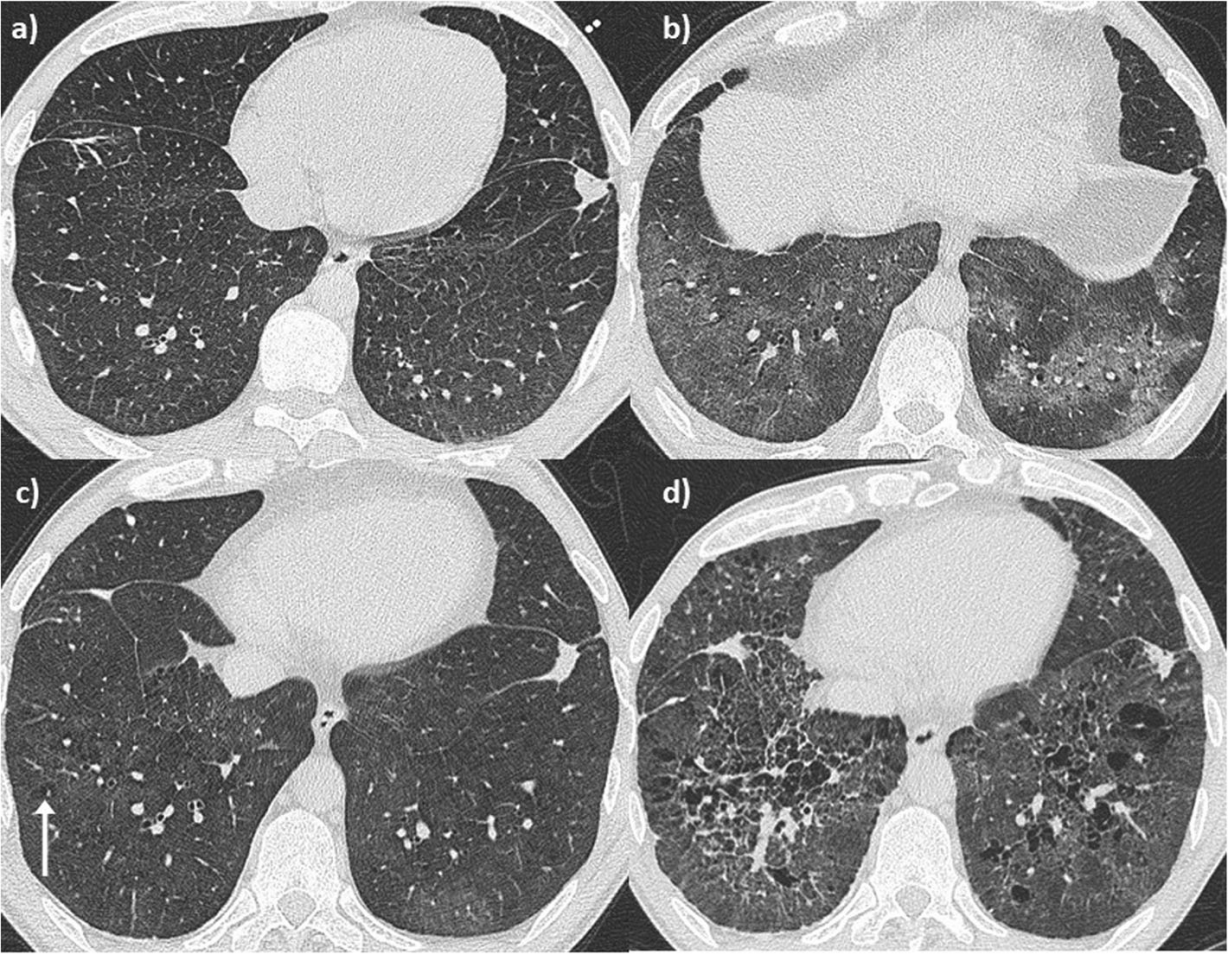
Patient 2. Radiological evolution. Axial CT images of the lung. **a** CT scan at first presentation (34 years old) demonstrates a subtle ground glass infiltrate, smooth interlobular septal thickening and a focal nodule in the subpleural left lower lobe. **b** At 40 years old, imaging reveals a more obvious ground glass infiltrate. **c** At 42 years old, perivascular emphysema/cysts are evident (white arrow), which **d** subsequently progresses to emphysema 2 years later. Similarly to Fig. 1 d this has a perivascular distribution

**Fig. 4:**
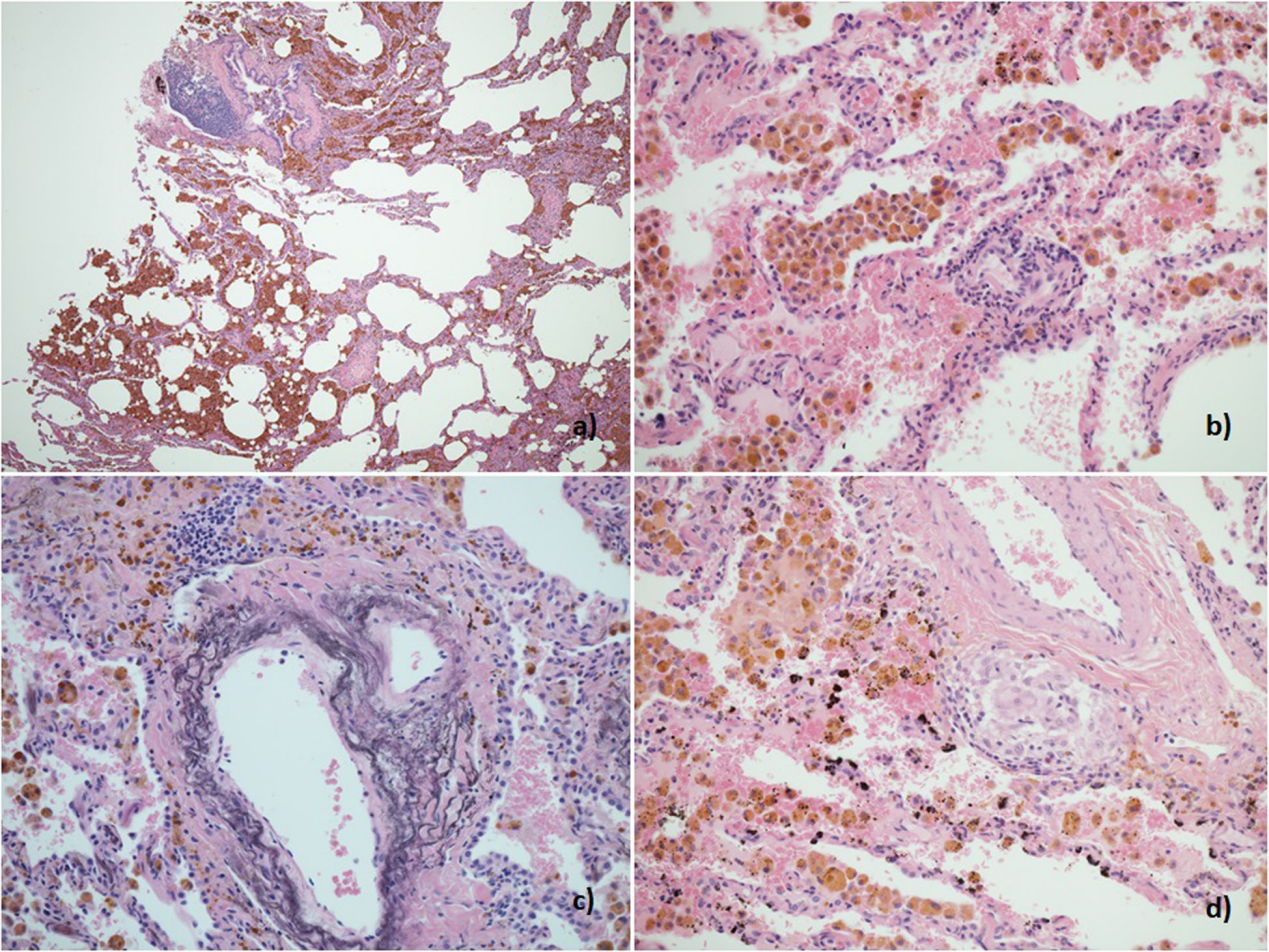
Patient 2. Histological sample, lung biopsy. **a** Marked accumulation of haemosiderin laden macrophages in alveolar spaces (H&E × 40). **b** Haemosiderin laden alveolar macrophages and focal perivascular lymphoid infiltrates (H&E × 200). **c** Iron encrustation of elastin fibres of small artery (H&E × 200) **d** Small perivascular non-necrotising granuloma (H&E × 200)

A 34-year-old man was referred to the RBH ILD unit with a two-year history of breathlessness on exertion and recurrent haemoptysis and anaemia requiring blood transfusions. Initial investigations at his local hospital included a chest HRCT scan showing diffuse ground glass opacity (Fig. 3 a), a negative autoimmune screen, normal lung function tests and a surgical lung biopsy histologically characteristic of DAH (Fig. 4). The patient was a farm tractor driver and his job involved spraying farming chemicals and working in grain stores, although he always wore a protective mask. He was an ex-smoker, having stopped 10 years prior to presentation (5 pack-year). He denied CTD symptoms. A repeat autoimmune screen including ANCA was negative apart from mild ANA positivity with a speckled pattern. Remaining blood tests revealed hypochromic microcytic anemia, and positive anti-thyroid antibodies. While positive anti-transglutaminase IgA antibodies were found, gastroenterology review including duodenal biopsies excluded coeliac disease. On BAL, Pearls stained pigmented macrophages were seen. Lung function tests were characterized by normal spirometry and mild gas transfer reduction (FEV1 127%, FVC 120.5%, TLC 106%, TLCO 84% and KCO 76%). He was started on low dose prednisone with resolution of the haemoptysis. He remained functionally and clinically stable for several years, slowly tapering the prednisolone dose. However, 6 years later he started complaining of episodic small volume haemoptysis and arthralgia, responsive to short courses of increased dose prednisol one. A repeat HRCT revealed more obvious ground glass infiltrates (Fig. 3 b). Eight years from first presentation p-ANCA with MPO positivity was identified (14 u/ml), confirmed on a second test (12 u/ml). In view of ongoing episodic small volume haemoptysis, methotrexate was added to background low dose prednisolone. Despite relatively good control of haemoptysis, with only occasional episodes consistently responsive to increased short course of prednisolone, eight years after his initial presentation, minor areas of emphysema began to appear on HRCT (Fig. 3 c) which progressively worsened becoming more evident in HRCT 2 years later. Perivascular emphysema was again observed (Fig. 3 d). In parallel with CT changes, a progressive worsening in his gas transfer was observed, with essentially stable spirometry but TLCO of 41% and KCO of 42% and normal echocardiographic evaluation.

### Patient 3

**Fig. 5:**
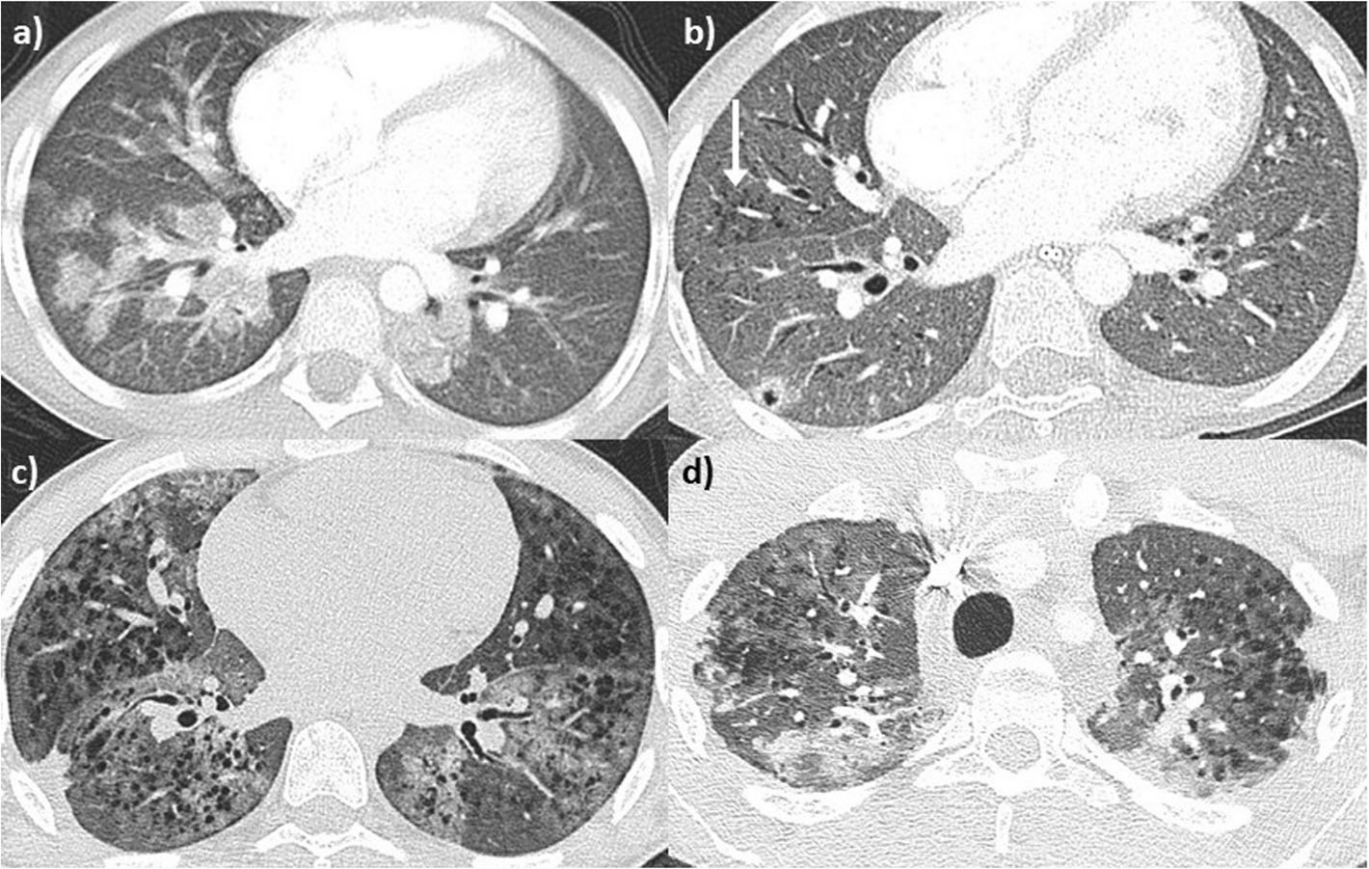
Patient 3. Radiologic evolution. Axial CT images of the lungs; **a**: At 9 years old the patient presented with an acute flare, post-contrast CT demonstrates ground glass nodules which resolved on follow up chest radiographs (not shown); **b**: At 11 years old a cavitating pulmonary nodule is present in the right lower lobe, and emphysema has developed surrounding the pulmonary vessels (arrow). The pulmonary nodule resolved on interval CT (not shown); **c**: At 15 years old the emphysema has proliferated, with a perivascular component again present. An acute ground glass infiltrate is also present; **d**: At 20 years old, peripheral polygonal areas of consolidation are present within the peripheral pulmonary parenchyma/pleura, in keeping with pleuroparenchymal fibroelastosis. A ground glass infiltrate is also present, as is emphysema

A 16-year old young man was transitioned to the Royal Brompton ILD service from the paediatric respiratory department with a diagnosis of IPH. He had the first episode of haemoptysis aged 7 and since then experienced recurrent episodes of haemoptysis, shortness of breath and cough. He was given a diagnosis of IPH based initially on symptoms, recurrent shadowing on imaging (Fig. 5 a) and negative autoimmune screen. Interestingly, aged 6 he was diagnosed with Hashimoto thyroiditis. Exacerbations of his IPH were strongly associated with flare ups of his thyroiditis. Until the age of 10, his symptoms and imaging findings responded to intermittent courses of oral corticosteroids of various doses.

**Fig. 6:**
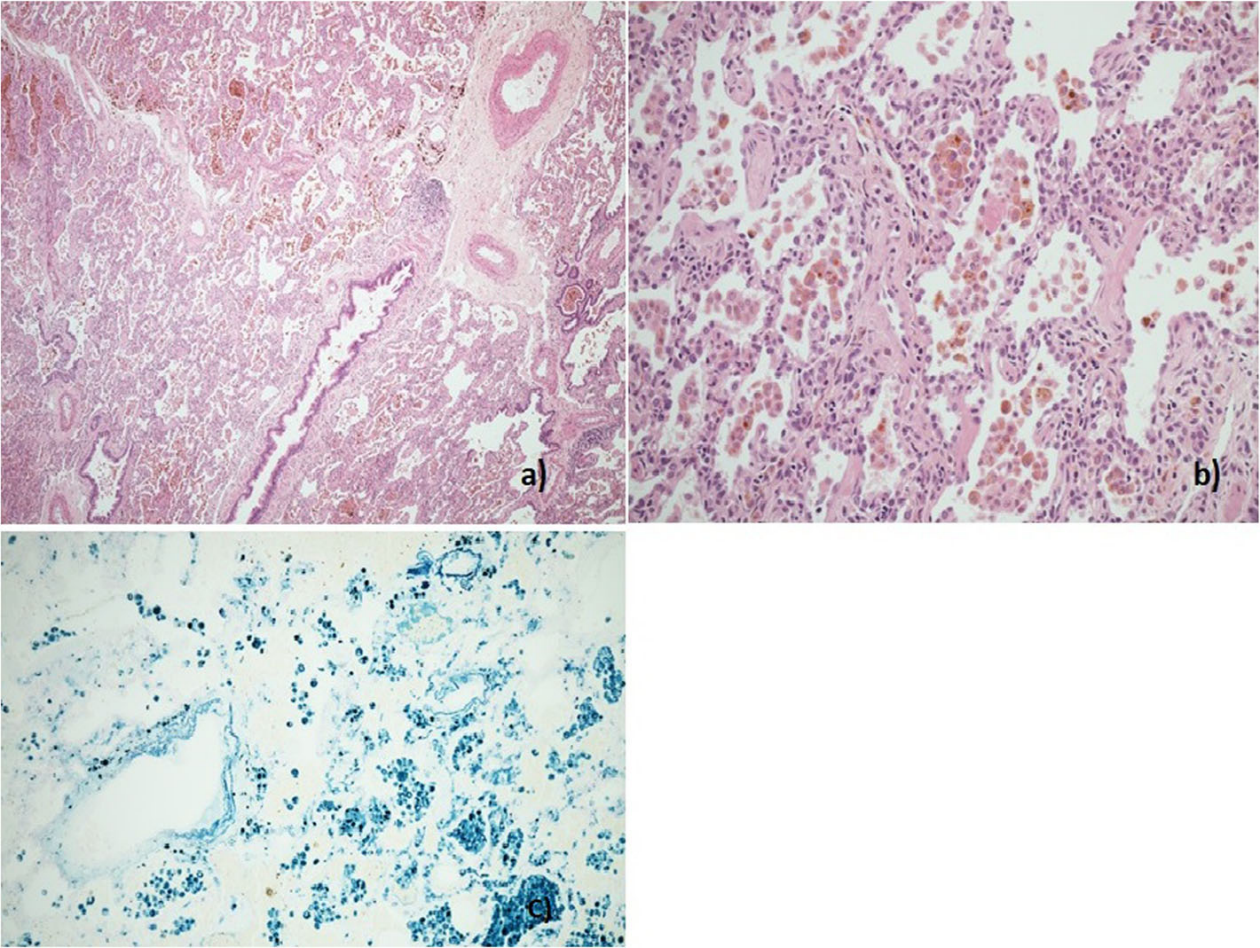
Patient 3. Histological sample, lung biopsy **a** Surgical lung biopsy showing marked accumulation of pigmented alveolar macrophages with scattered small lymphoid aggregates (H&E × 100). **b** Alveolar macrophages with coarse golden-brown cytoplasmic pigment. There is reactive type 2 hyperplasia and mild expansion of alveolar walls with scattered small lymphocytes and minimal fibrosis, but no features of capillaritis or vasculitis are seen (H&E × 200). **c** The cytoplasmic pigment is positive with Perls Prussian Blue stain confirming it to be haemosiderin. Note also iron encrustation of elastin fibres in small vessels (Perls Prussian Blue stain × 200)

Aged 11 he was admitted to the local hospital with haemoptysis and severe respiratory failure. He was intubated and transferred to the paediatric intensive care unit at RBH. He underwent BAL and surgical lung biopsy. The BAL revealed large numbers of haemosiderin-laden macrophages on Perl’s stain. The biopsy showed DAH without supporting evidence of vasculitis (Fig. 6). His HRCT at the time of the biopsy demonstrated diffuse ground glass opacity, perivascular emphysema and a small cavity in the right lower lobe (Fig. 5 b) which raised suspicion for vasculitis but repeat ANCA testing was negative. He was treated with antibiotics and intravenous corticosteroids with good response and resolution of the cavity on subsequent imaging. His spirometry prior to this acute episode showed FEV1 41% and FVC 37%.

Upon discharge, he was commenced on maintenance treatment with hydroxychloroquine, low dose prednisolone and azithromycin 250 mg three times weekly. Short term courses of increased doses of prednisolone were used for flare ups. A repeat HRCT 4 years after the biopsy showed extensive emphysema with a perivascular distribution, and superimposed diffuse ground glass opacities (Fig. 5 c). The patient had never smoked nor had he ever been exposed to passive smoking.

When he transitioned to the adult ILD clinic and 8 years after the biopsy, aged 19, the radiological picture had further progressed and was characterized by extensive emphysema and pleuroparenchymal fibroelastosis (PPFE) of both upper lobes (Fig. 6 d). Lung function was severely impaired with FEV1 54%, FVC 46%, FEV1/FVC 99%, TLCO 33% and KCO 68% reflecting the combination of severe emphysema and PPFE. A repeat autoimmune screen revealed a p-ANCA with MPO positivity (4.6 u/mL). Following these results, he was started on low dose azathioprine, with ongoing hydroxycloroquine and azithromycin, with current symptomatic and functional stability.

## Discussion and conclusions

Here we present three cases of chronic DAH initially diagnosed as IPH that subsequently developed ANCA-MPO positivity, and, on HRCT, progressive development of cystic areas in keeping with emphysema, all with a striking perivascular distribution. We believe these three cases are of interest in highlighting the 1) possibility of development of ANCA +/− renal involvement several years down the line from first IPH presentation; 2) the possibility that DAH changes may develop into emphysema.

### Development of ANCA MPO antibodies

The development of specific antibodies +/− other typical autoimmune extrapulmonary manifestations after the initial pulmonary presentation have been described in a number of scenarios [3–6]. Specifically, to the development of ANCA MPO positivity, longitudinal assessments have revealed new onset of MPO+ or PR3+ and/or overt vasculitis on follow up in patients initially presenting as idiopathic interstitial pneumonias [3].

However, there are only a few reports detailing subsequent autoimmune serology (other than ANCA) conversions following an initial diagnosis of IPH [7, 8]. Le Clainche et al. [4] report on 15 children diagnosed with IPH, of which only one developed ANCA on follow up (specificity not reported). However, as the children were not screened for ANCA at presentation, it is impossible to conclude whether this was a real seroconversion.

ANCA MPO seroconversion occurring in adulthood in IPH after many years has been described, to our knowledge, only in another case [5]. However, the patient presented by Freitas et al. was a 21 year old girl diagnosed with IPH at the age of 4, who developed ANCA positivity 12 years after presentation, aged 16, and had a good response to immunosuppression. Furthermore, no emphysematous changes on follow up were mentioned. By contrast, our cases developed ANCA positivity in adulthood, and all continue to require ongoing immunosuppressive treatment to control the DAH, suggesting the possible role played by autoimmune phenomena in this rare disease, even when initially deemed “idiopathic”. In support of this, the second case had positive anti-transglutaminase IgA antibodies and autoimmune thyroiditis and the third case had autoimmune thyroiditis.

### Development of emphysema

During follow up, our cases developed cystic opacities consistent with emphysema, not seen at presentation. In the first patient, limited emphysema was first seen 10 years after presentation. The cystic areas became more prominent over the following 6 years, with concurrent isolated reduction in DLCO. The second patient was first noted to have developed emphysematous changes on HRCT 8 years after the initial presentation, and 20 years after having completely stopped smoking, with further significant worsening of radiological emphysema after another 2 years. In parallel, there was marked worsening in gas transfer with persistently normal lung volumes. The third patient was diagnosed with IPH in early childhood and was first noted to have emphysema on HRCT 4 years after the diagnosis, aged 11, with subsequent progression despite the lack of active or passive smoking exposure.

The development of emphysema in the context of interstitial processes even outside of a significant history of smoking is being increasingly recognized. In a cohort of never-smoker patients with rheumatoid arthritis related ILD, 27% had associated emphysema with obstructive functional indices [6], while in systemic sclerosis related ILD patients with concomitant emphysema, the prevalence of never smokers was 33% [9]. In a cohort of 233 hypersensitivity pneumonia (HP) patients evaluated for the presence of emphysema and PPFE, 23% of patients with emphysema were never-smokers [7].

In AAV, development of emphysema has been described in a few retrospective case series and case reports [8, 10–12]. In a cohort of AAV patients with 79 granulomatosis with polyangiitis (GPA) cases and 61 microscopic polyangiitis (MPA) cases, global emphysematous changes incidence was 13% with no significant differences between the two groups [12]. In the largest published series so far, Yagamata et al. reported emphysematous lesions in 37% of patients in a cohort of MPA patients, more frequently characterized by low attenuation areas rather than cystic lesions, although the Authors did not link the emphysematous lesions with alveolar haemorrhage per se [13].

The pathogenesis of emphysema on a background of DAH and ANCA positivity is unclear. One possibility is that the emphysema seen in our three cases could be secondary to the inflammatory process present within the capillaries/small vessels leading to the destruction of the alveolar walls though the release of noxious insults including proteolytic enzymes and free oxygen radicals. The observation of a striking peribronchovascular distribution to the emphysema supports this hypothesis. This has been suggested in the context of capillaritis [14] and in hypocomplementemic urticarial vasculitis, a rare immune complex related small vessel vasculitis, characterized by low C1q and C4 complement levels. Serial histopathological analysis of one case described by Hunt et al. [15] showed that the initial changes of capillaritis evolved into emphysema, and obstructive airways disease. Several other case reports have presented this association in the context of hypocomplementemic urticarial vasculitis [16–22].

It is also possible that the ANCA antibodies themselves have pathogenic activity. Neutrophils can be activated by MPO-ANCA causing the release of MPO, a cationic protein expressed by several immune cells including neutrophils and macrophages, resulting in an inflammatory response [23]. In an animal model of smoke-induced emphysema, a myeloperoxidase inhibitor prevented progression of the emphysematous changes [24] Further support for a role played by autoantibodies and specifically ANCA, comes from the finding of significantly increased proportion of combined pulmonary fibrosis an emphysema (CPFE) among patients with ANA positivity and a higher MPO-ANCA positivity compared to idiopathic pulmonary fibrosis (IPF) patients without emphysema. Furthermore, CPFE patients with ANA and/or ANCA positivity showed an increased number of CD20+ cells forming lymphoid follicles within the fibrotic interstitium in areas adjacent to fibroblastic foci [25].

In the first two cases, we cannot completely exclude that the development of cystic changes and/or emphysema was related to the previous history of smoking, and certainly cigarette smoking may have contributed to its development. However, the first case had a truly trivial smoking history (one pack year), and stopped by age 21, when her HRCT showed no cystic/emphysematous lesions. The second patient had a 5-pack year history but he stopped and had been a non-smoker for 10 years by the time he had his initial HRCT, which did not reveal any emphysematous destruction. Furthermore, the third patient developed extensive emphysema at a very young age with no exposure to cigarette smoke, supporting a direct link between DAH, ANCA positivity and emphysema.

Further studies are required to better understand the relationship between DAH, ANCA positivity and development of emphysema. ANCA positivity seems to be retrospectively associated with a worse outcome in children with IPH [26]. Also, the development of emphysema in the contest of CTDs and HP has been associated to a worse prognosis [6, 7, 9].

IPH is a rare disease and little is known about the mechanisms involved in its development. However, its known association with coeliac disease [27], its response to immunosuppressive therapy and its possible association with autoimmune features [4, 28, 29] suggests that a contribution of immunological overactivity, even if a clear cut autoimmune disease is not identified.

Immunosuppressive treatment should always be considered in these patients even in the context of an idiopathic onset. Patients with IPH should have ANCA screening not only at presentation but also during follow up, especially when the response to therapy is poor or in presence of suspected renal involvement. However, it is worth noting that ANCA positivity is not always identified in AAV, such that the presence of ANCA is not essential to confirm the diagnosis or to start immunosuppressive therapy, if needed [30]. Full lung function tests including DLCO should be monitored frequently even in case of stable spirometric values, as subtle worsening due to development of emphysematous changes could develop even after many years. This could aid in identifying the cases which could benefit from a more aggressive therapy and perhaps prevent the development of complications such as emphysematous changes.

## Data Availability

Data sharing is not applicable to this article as no datasets were generated or analysed during the current study.

## Abbreviations

ANA: Anti-nuclear antibodies
ANCA: Anti-neutrophil cytoplasmic antibody
anti-ds DNA: Anti-double-stranded DNA
anti-GBM: Anti–glomerular basement membrane
BAL: Bronchoalveolar lavage
CPFE: Combined pulmonary fibrosis and emphysema
CT: Computed tomography
CTD: Connective tissue disease
DAH: Diffuse alveolar haemorrhage
ECG: Electocardiogram
eGFR: Estimated glomerular filtration rate
ENA: Extractable nuclear antigens
FEV1: Forced expiratory volume in 1 s
FVC: Forced vital capacity
GPA: Granulomatosis with polyangiitis
HP: Hypersensitivity pneumonitis
HRCT: High resolution computed tomography
ILD: Interstitial lung disease
IPF: Idiopathic pulmonary fibrosis
IPH: Idiopathic pulmonary hemosiderosis
KCO: Transfer coefficient
MPA: Microscopic polyangiitis
MPO: Anti-myeloperoxidase antibodies
MRC: Medical Research Council
PPFE: Pleuroparenchymal fibroelastosis
PR3: Anti-proteinase 3 antibodies
RBH: Royal Brompton Hospital
RF: Rheumatoid factor
TLC: Total lung capacity
TLCO: Carbon monoxide transfer factor
